# Supplementary figures and images for: A simulation study investigating power estimates in phenome-wide association studies
Source: BMC Bioinformatics. 2018 Apr 4;19:120. doi: 10.1186/s12859-018-2135-0 (PMC5885318; doi:10.1186/s12859-018-2135-0)

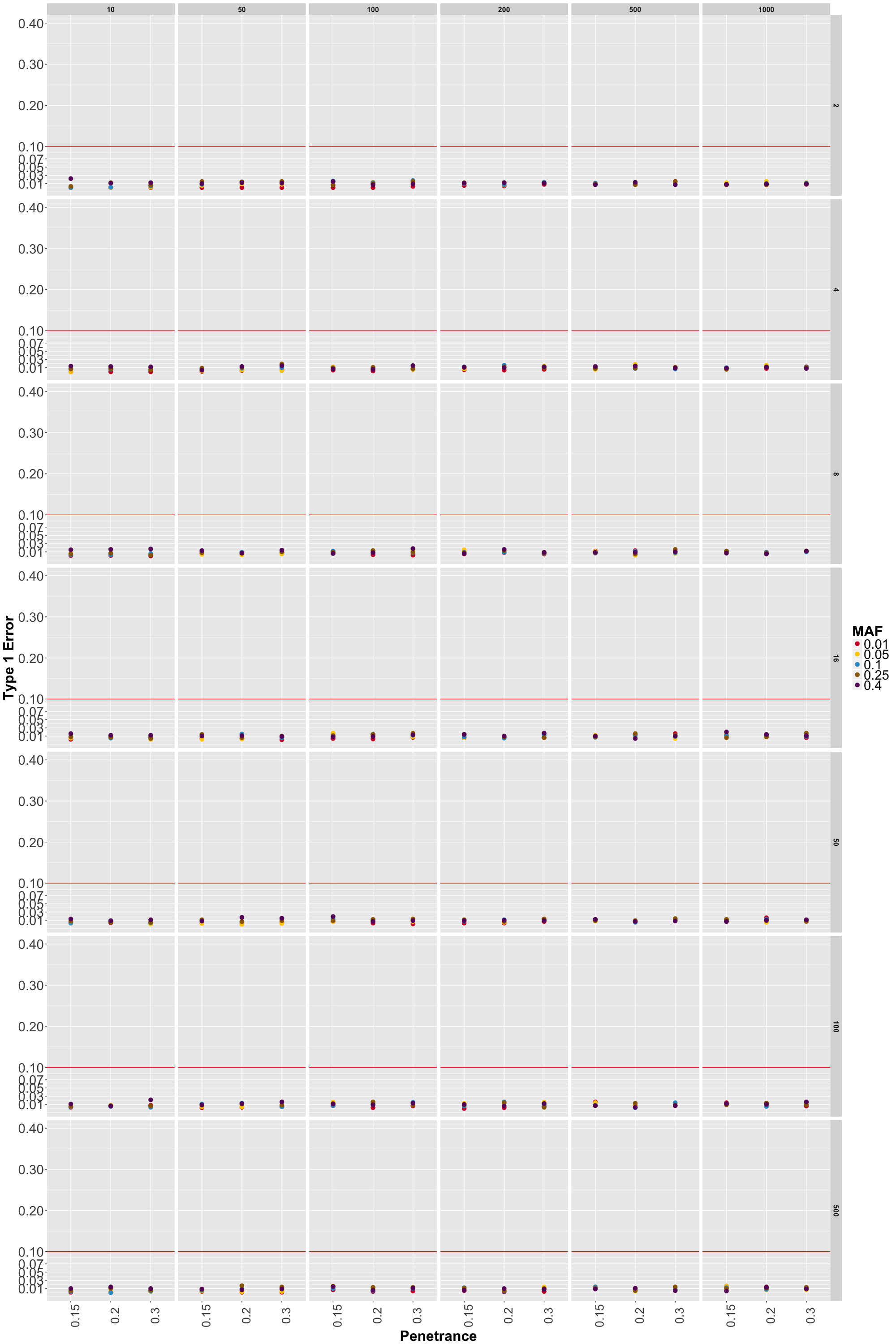

Supplement: Supplementary file 1 — Figure S1. Binary Trait Type I Errors. The plot shows the Type I errors for different parameter settings. Each panel represents the different case number on the top and case-control ratio on the right which was used for the simulation dataset. The Type I error on the y-axis is calculated based on the number of false positive association below significance level of α = 0.00025. The disease penetrance is represented on the x-axis and each colored point represent different MAF used in the simulations. (PNG 379 kb) [file 12859_2018_2135_MOESM1_ESM.png]
